# Heated tobacco products- well known or well understood? A national cross-sectional study on knowledge, attitudes and usage in Pakistan

**DOI:** 10.1186/s12889-024-18825-y

**Published:** 2024-05-16

**Authors:** Hammad Atif Irshad, Hamzah Jehanzeb, Sajjan Raja, Umair Saleem, Wamiq Ali Shaikh, Akmal Shahzad, Atiqa Amirali, Nousheen Iqbal, Javaid Ahmed Khan

**Affiliations:** 1 Medical College, Aga Khan University, Karachi, 74800 Pakistan; 2 Jinnah Medical and Dental College, Karachi, 74800 Pakistan; 3 Section of Pulmonary and Critical Care Medicine, Department of Medicine, Aga Khan University Hospital, Karachi, 74800 Pakistan

**Keywords:** Heated tobacco products, Non-cigarette, Public health, Knowledge

## Abstract

**Background:**

Heated tobacco products (HTPs) are reshaping the tobacco industry and just recently, a plan was sought to regularize HTPs in Pakistan. Pakistan provides an intriguing case study in this context, as tobacco use is deeply ingrained in public use. To ensure that future evidence-based policy recommendations are grounded in the public’s knowledge, attitudes, and usage of HTPs, a nationwide survey must be conducted.

**Methods:**

A cross-sectional study was conducted using an online-based questionnaire nationwide in Pakistan. The questionnaire was validated and distributed through convenience sampling. The questionnaire assessed participants’ knowledge, attitudes, and usage of HTPs. Descriptive statistics was used to describe participants’ response and linear regression was performed at a *p*-value of < 0.05 using SPSS version 26.

**Results:**

In our sample of 1195 respondents (mean age of 33 years, 41.8% males and 58.2% females), 54.7% had previously heard about HTPs and 16.9% reported using HTPs at least once. Additionally, 38.24% were unsure of the legality of HTP use. Those with monthly household incomes of PKR 100,000 to 500,000, were more likely to have higher knowledge scores (OR:1.80[1.07–3.04]). On the other hand, males (OR:0.70 [0.55–0.89]) and respondents from Balochistan (OR:0.40 [0.22–0.71]) were more likely to have lower knowledge scores. The strongest motivators were the enjoyability of HTPs (55.73%) and usage as a cigarette alternative (54.64%), while the strongest deterrents were the negative health effects (82.68%) and potential for addiction (81.01%).

**Conclusion:**

Our study underscores the need for awareness campaigns and interventions concerning HTPs, given prevalent preconceived notions and mixed attitudes among respondents. It was found that women and households with higher incomes scored higher on knowledge. Subjective enjoyment and a substitute for cigarettes were important motivators, but the most mentioned deterrents were the possibility of addiction and the detrimental effects on health. These insights form the basis for informed policy making for non-cigarette tobacco products.

## Introduction

Heated tobacco products (HTPs) are electronic devices designed to warm tobacco to a specific temperature, around 350 degrees Celsius to produce aerosol without burning or generating smoke, which typically occurs at about 650 degrees Celsius during ignition [1]. This characteristic has led to this product being referred to as “heat-not-burn” products in the tobacco industry [1]. While a variety of products are available that accomplish the heating through slightly different methods, most of them come with a holder that is used in conjunction with processed tobacco in the form of sticks or capsules [2]. On the other hand, tobacco is not used in electronic cigarettes (e-cigarette) [3]. When a user draws on an e-cigarette, however, they vaporize an e-liquid solution that contains flavors and nicotine [3].

Tobacco companies have marketed HTPs and e-cigarettes as substitutes for traditional cigarettes that burn [4]. This has been carried out in addition to the creation of specialty stores with competitive pricing in comparison to other tobacco products and the promotion and advertising of HTPs through a variety of media channels [5]. HTP companies anticipate a substantial global market value nearing USD 68 billion by 2027 [6, 7], representing a seven-fold increase from 2020. The expected surge in buying is likely due to increasing popularity among young adults and both current and former smokers, raising concerns from public health officials on HTPs serving as an entry into tobacco use [8–10]. This concern is further burdened by the uncertainty revolving around the immediate and long-term risks of HTP use [11].

In this evolving landscape, Pakistan emerges as a distinctive and dynamic setting for comprehensive exploration of public perception. The nation’s sociodemographic landscape is characterized primarily by its youthful population, coexisting with substantial rates of smoking tobacco habits [12].

According to a 2022 statistic, there are an estimated 25.4 million adults aged 15 and over who use tobacco products in Pakistan, leading to a 7th ranking globally and 1st in the WHO Eastern Mediterranean Region [13]. At the same time, marketing for tobacco products remains unregulated, with products being sold both online and in stores without any restrictions or bans [14]. These complexities are further compounded by challenges in effectively implementing tobacco control measures [15, 16].

Previous studies on other alternatives to traditional cigarettes in Pakistan revealed varying perceptions; some viewed them as less harmful and potentially helpful for quitting smoking, while others raised concerns about chronic diseases and addiction [17]. A study conducted among teenagers in Pakistan in revealed that nearly half of them cited peer pressure or a desire to quit smoking conventional cigarettes as reasons for using e-cigarettes [18]. Additionally, approximately one-third strongly believed that using e-cigarettes poses self-harm risks and that they are as harmful and addictive as regular cigarettes [18]. Although, no study gauging perceptions regarding HTPs exists in Pakistan, a survey of adults in the United States showed varying perceptions about IQOS, a type of heated tobacco product with only a quarter of participants agreeing it was socially acceptable, while 15.4% thought it was FDA-approved as safe and 15.0% believed it was less addictive than cigarettes [19]. Furthermore, in Japan, even healthcare practitioners have revealed a gap in knowledge regarding HTPs, further emphasizing the need for tailored awareness campaigns in the unique Pakistani context with its distinct cultural and tobacco usage landscape [20].

Public surveys are vital for gauging public opinion and implementing impactful policy changes [21]. However, no past study in Pakistan has evaluated public opinion for HTPs. Therefore, the aims of our study are to assess the knowledge, attitudes, and usage of the public in Pakistan towards a relatively new tobacco products, heated tobacco products. Through this we aim to provide a foundation for developing targeted educational strategies and refining healthcare practices, thereby advancing public health outcomes.

## Methods

### Study design, setting and population

This cross-sectional study was conducted across Pakistan through a web-based questionnaire. The target population for this questionnaire was adult individuals (age ≥ 18 years) residing within Pakistan.

### Data collection tool and variables

In the absence of a prior questionnaire suitable for our population, a comprehensive questionnaire was developed using elements from the Global Adult Tobacco Survey (GATs) and other tools which have previously been utilized within Pakistan [22–25]. The English questionnaire was also translated to Urdu, which is the national language of Pakistan, by an independent translator fluent in both languages and with experience in questionnaire translation.

The final components of the questionnaire were rigorously reviewed in close association with faculty experts in tobacco research at the Section of Pulmonary and Critical Care Medicine at AKUH. Content validity was assessed by calculating a content validity index (CVI) for this survey for relevance, essentiality and clarity based on the ratings of two subject experts. A CVI of 0.942, 0.942 and 0.8986 was calculated for these three parameters respectively, which was deemed acceptable according to Davis (1992) [26]. Furthermore, to ensure face validity, the English and Urdu versions of the questionnaire underwent pilot testing amongst 50 respondents. A 7-item knowledge, 27- item attitude and 5 item usage questionnaire was used for pilot testing. Cronbach’s alpha value was calculated for multiple-choice questions in each of these domains to check for internal consistency, with the calculated values being 0.8597, 0.9503 and 0.86 for the knowledge, attitudes, and usage domains respectively. These scores reflected good, excellent, and good consistency respectively.

The final survey contained four sections (Demographics, Knowledge, Attitudes and Usage) and was preceded by a consent form explaining the nature and scope of the survey.

### Data collection procedure

Responses were collected by means of a questionnaire that was available in both English and Urdu, the national language of Pakistan. Our questionnaire was circulated amongst adults (age ≥ 18) as a Google Form, and on social media platforms, such as WhatsApp groups and Facebook groups.

### Sampling and study size calculation

For this web-based study, convenience sampling and snowball sampling methods were utilized to reach adults across the country. Convenience sampling was our primary technique, allowing participants to easily access and complete the online survey. Additionally, snowball sampling was employed, where initial participants were asked to refer others to participate. These methods facilitated the collection of diverse perspectives from across the country.

The sample size was calculated using OpenEpi [27]. According to the Pakistan Demographic and Health Survey 2017-18, the current smoking prevalence among men ages 15–49 is 31.8% [28]. Using that statistic, at least 334 participants aged 18 years and above were determined to be recruited. An additional 10% margin of error was taken for a minimum required sample size of 367.

### Statistical analysis

Statistical analyses were run using Statistical Package for Social Sciences (SPSS) version 26 [29]. Continuous data such as age was reported using mean and standard deviation. Categorical data consisting of the results of the Likert scales and multiple-choice questions have been reported as frequencies and percentages. To analyze the effect of factors such as age, sex, and level of education on the overall knowledge score, a linear regression was performed. A *p*-value of < 0.05 was considered significant for all analyses.

## Results

Responses were collected from a total of 1,195 participants. The mean age of the participants was 33.17 ± 3.70, with 41.6% being male and 58.4% female. The majority of respondents resided in provinces of either Sindh (42.09%) or Punjab (32.30%), while Karachi (36.32%) and Lahore (10.88%) were the most represented cities. Additionally, most participants reported visiting a doctor or healthcare provider 1–2 times in the past 12 months (50.63%). Demographic characteristics of the sample have been summarized in Table 1.

**Table 1:** Demographics characteristics

Variable	N (%)
**Mean Age ± SD**	33.17 ± 3.70
**Sex**	
Male	499 (41.6)
Female	696 (58.4)
**Province of Residence**	
Punjab	386 (32.30)
Sindh	503 (42.09)
Islamabad	48 (4.02)
Balochistan	52 (4.35)
Khyber Pakhtunkhwa	176 (14.73)
Azad Jammu and Kashmir	25 (2.09)
Gilgit Baltistan	5 (0.42)
**City of Residence**	
Karachi	434 (36.32)
Lahore	130 (10.88)
Peshawar	128 (10.71)
Bahawalpur	65(5.44)
Quetta	51(4.27)
Rawalpindi	46 (3.85)
Islamabad	57 (4.77)
Hyderabad	33 (2.76)
Tando Allahyar	13 (1.09)
Other	238(19.92)
**Level of Education**	
Primary school	1 (0.08)
Matric/ O level	9 (0.75)
Intermediate/ A level	124 (10.38)
Bachelor’s Degree	748 (62.59)
Postgraduate Degree	312 (26.11)
No formal education	1 (0.08)
**Marital Status**	
Unmarried	948 (79.33)
Married	241 (20.17)
Divorced	4 (0.33)
Widowed	2 (0.17)
**Monthly Family Income (PKR)**	
<25,000	69 (5.77)
25,000–50,000	118 (9.87)
50,000-100,000	319 (26.69)
100,000-500,000	517 (43.26)
>500,000	172 (14.39)
**How many times have you visited a doctor or health care provider in the past 12 months?**	
0	272 (22.76)
1 or 2	605 (50.63)
3 to 5	218 (18.24)
6 or more	100 (8.37)

PKR: Pakistani Rupees

SD: Standard Deviation

Table 2 depicts the knowledge of the participants towards HTPs. A majority of participants, 54.73%, reported having heard of HTPs prior to the survey. When asked about the contents of these products, 49.96% believed they contained both nicotine and tobacco, while 27.78% thought they contained only nicotine. Notably, 40.25% thought these products were not prohibited by law and 38.24% were unsure if these products were prohibited by law. Regarding health risks, 68.37% agreed that these products can cause serious illnesses like cancer and stroke. Additionally, 70.38% believed these products had added chemicals. Lastly, a significant portion, 58.83%, strongly disagreed that these products are safe to use during pregnancy, and 58.91% strongly disagreed that they are safe for individuals with underlying medical conditions such as heart disease, blood pressure, and diabetes.

**Table 2:** Knowledge regarding HTPs

Variable	*N*(%)
**Before this survey, had you heard about HTPs?**	
Yes	654 (54.73)
No	541 (45.27)
**What do you think is contained in HTPs?**	
Nicotine	332 (27.78)
Tobacco	100 (8.37)
Nicotine plus tobacco	597 (49.96)
Neither	79 (6.61)
Other	87 (7.28)
**Prohibited by law**	
Yes	257 (21.51)
No	481 (40.25 )
Maybe	457 (38.24)
**Cause serious illness e.g., cancer, stroke.**	
Strongly Disagree	83 (6.95)
Disagree	62 (5.19)
Neutral	233 (19.50)
Agree	419 (35.06
Strongly Agree	398 (33.31)
**Have added chemicals.**	
Strongly Disagree	53 (4.44)
Disagree	85 (7.11)
Neutral	216 (18.08)
Agree	450 (37.66)
Strongly Agree	391 (32.72)
**Safe to use during pregnancy.**	
Strongly Disagree	703 (58.83)
Disagree	234 (19.58)
Neutral	168 (14.06)
Agree	41 (3.43)
Strongly Agree	49 (4.10)
**Safe to use in people with underlying medical conditions such as heart disease, blood pressure and diabetes.**	
Strongly Disagree	704 (58.91)
Disagree	219 (18.33)
Neutral	165 (13.81)
Agree	57 (4.77)
Strongly Agree	50 (4.18)

Linear regression analysis is seen in Table 3. Among the sociodemographic factors analyzed, three variables were statistically significant factors associated with higher knowledge scores. Firstly, males were significantly associated with having low knowledge score (*p* = 0.004) compared to females. Secondly, participants residing in Balochistan demonstrated significantly lower knowledge scores compared to those in Sindh (*p* < 0.001), indicating a regional disparity in tobacco-related knowledge. Secondly, participants with a monthly household income between 100,000 and 500,000 PKR exhibited higher knowledge scores (*p* = 0.026), suggesting that a relatively higher income is associated with greater awareness of tobacco products.

**Table 3:** Factors affecting Knowledge towards HTPs

Variable	OR	95% CI Low	95% CI High	p-value
**Age (years)**	1.00017	0.99927	1.00108	0.712
**Sex**				
Female	1 (reference)	-	-	-
Male	0.70308	0.55442	0.8916	0.004*
**Level of Education**				
No formal education	1 (reference)	-	-	-
<5 years (Primary school)	2.23984	0.00148	3389.08	0.829
5–10 years (Matric/ O level)	24.1156	0.06665	8725.83	0.289
10–12 years (Intermediate/ A level)	25.3681	0.08102	7942.71	0.27
12–14 years (Bachelor’s degree)	17.8156	0.05772	5498.5	0.324
>14 years (Postgraduate degree)	17.7361	0.05691	5527.24	0.326
**Marital Status**				
Unmarried	1 (reference)	-	-	-
Married	0.91544	0.66799	1.25457	0.582
Divorced	2.91414	0.28974	29.3095	0.363
Widowed	2.33533	0.04221	129.197	0.678
**Province of Residence**				
Sindh	1 (reference)	-	-	-
AJK	0.98772	0.4302	2.26775	0.977
Balochistan	0.39535	0.22046	0.70896	0.002*
Gilgit Baltistan	1.06072	0.17673	6.36619	0.949
Islamabad Capital Territory	0.77086	0.41983	1.41541	0.401
KPK	0.81349	0.57205	1.15685	0.25
Punjab	0.78257	0.59476	1.02968	0.08
**Monthly Household Income (PKR)**				
<25,000	1 (reference)	-	-	-
25,000–50,000	0.76255	0.41429	1.40355	0.384
50,000-100,000	1.0945	0.64036	1.87068	0.741
100,000-500,000	1.80463	1.07185	3.03839	0.026*
>500,000	1.4529	0.8142	2.59263	0.206
**Number of Doctor Visits in Past 12 Months**				
**0**	1 (reference)	-	-	-
1 or 2	0.97378	0.72731	1.30377	0.858
3 to 5	1.23836	0.86137	1.78035	0.248
6 or more	0.93467	0.58334	1.49761	0.779

Asterisk (*) denotes statistical significance

**Fig. 1:**
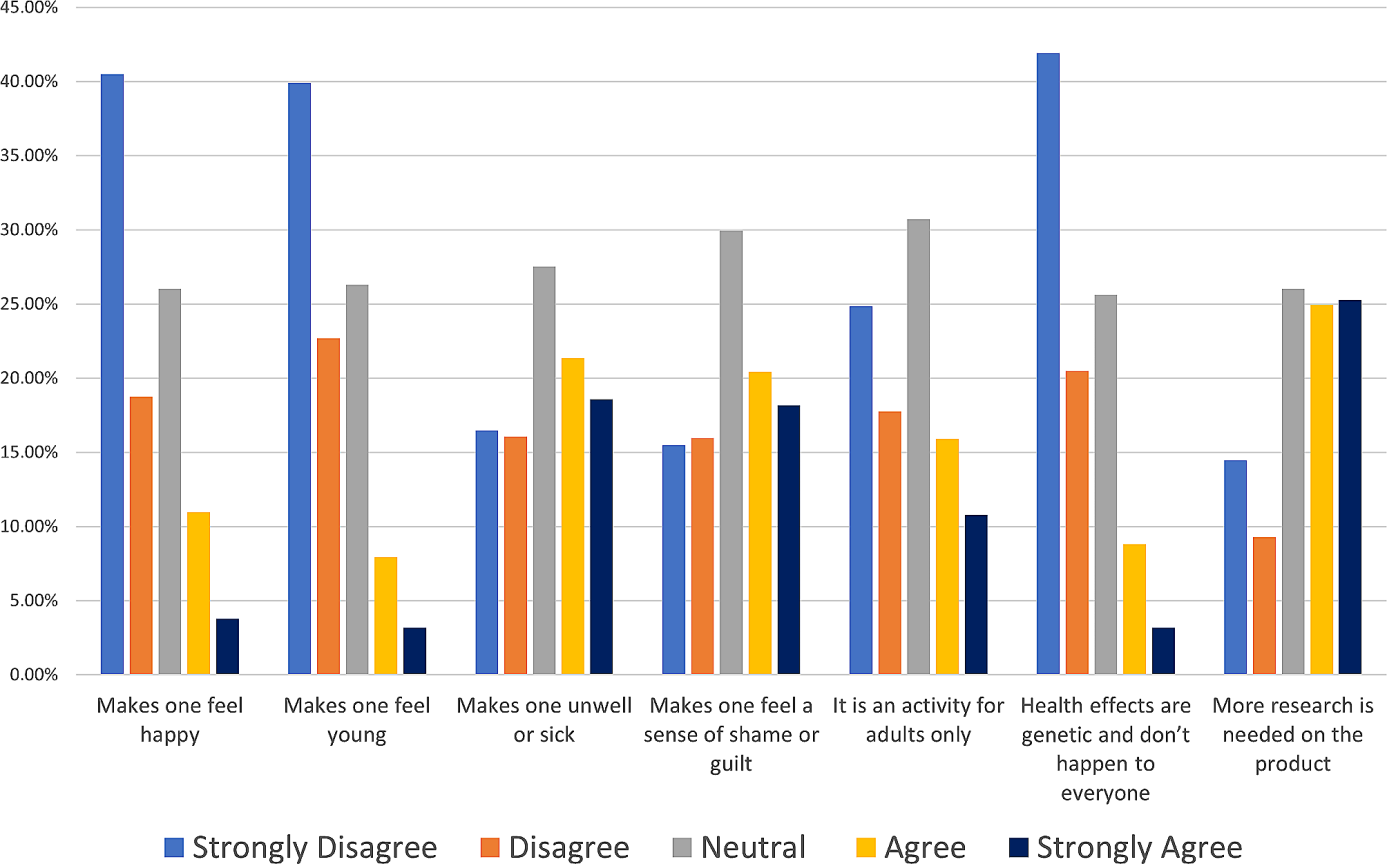
General attitude of the public towards HTPs

Figure 1 shows the general attitude of the participants towards HTPs. A substantial proportion, 62.26%, responded “don’t know” when asked about the satisfaction level of using these products compared to traditional cigarettes. When exploring the attitudes of our participants towards heated tobacco products (HTP), the majority disagreed or strongly disagreed that they make one feel happy (59.24%), young (62.60%), unwell or sick (32.56%), or evoke a sense of shame or guilt (31.46%). Interestingly, the majority also disagreed or strongly disagreed with the notion that these products are exclusively for adults (42.59%) and that health effects are genetic and don’t happen to everyone (62.42%). Additionally, a significant number of participants, 50.21%, believed that more research is needed on these products.

**Fig. 2:**
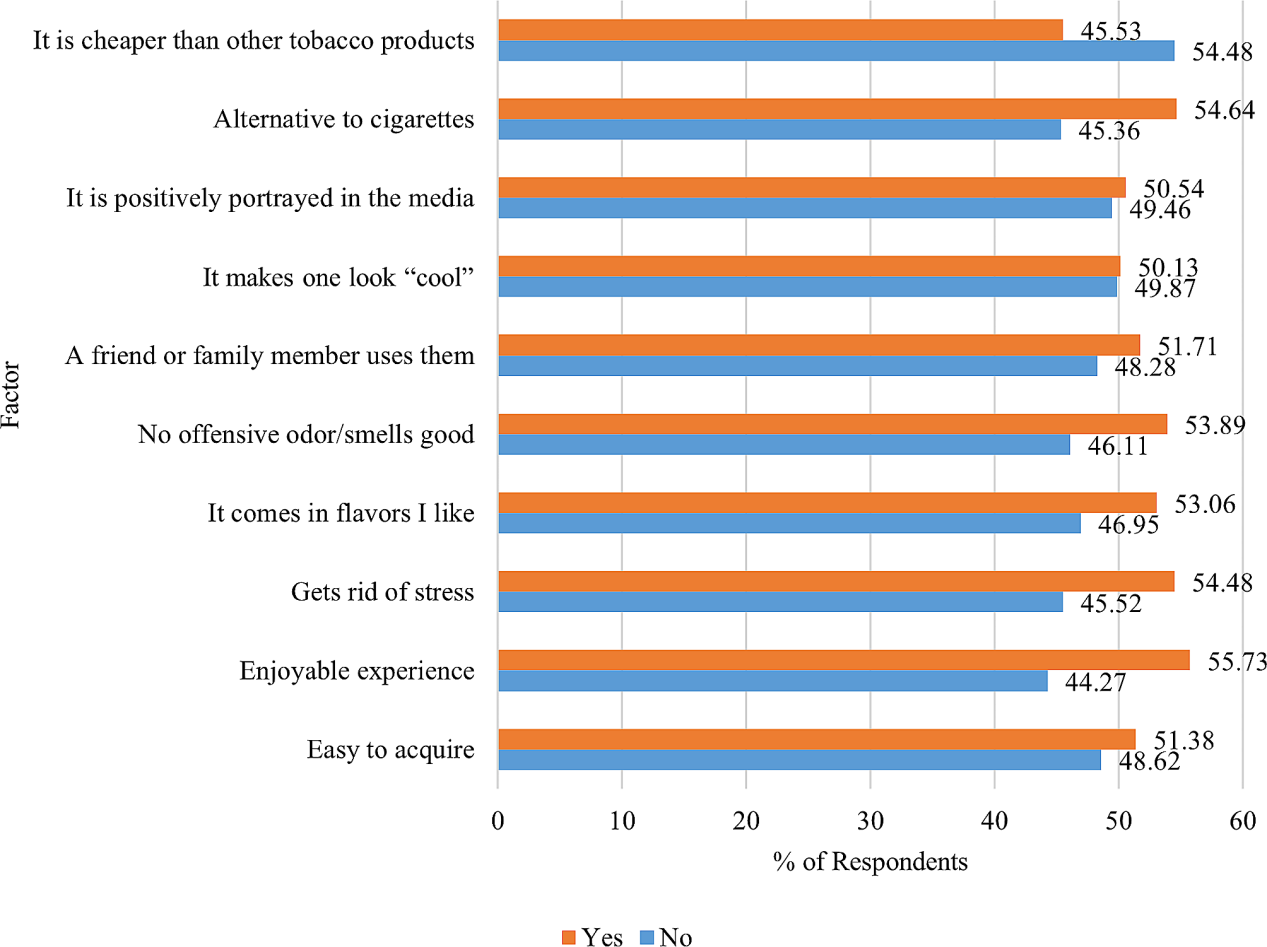
Factors motivating HTP use among respondents

Motivators to consider pursuing heated tobacco products (HTP) were also gauged as seen in Fig. 2. The most agreed motivator in our study was “enjoyable experience” (55.73%). Among other factors, respondents also agreed with the statement “alternative to cigarette” (54.64%) and “gets rid of stress” (54.48%). Most “No” responses in terms of motivating factor were received for HTPs being cheaper than other tobacco products (54.48%) and secondly, the statement of “it makes one look “cool””.

**Fig. 3:**
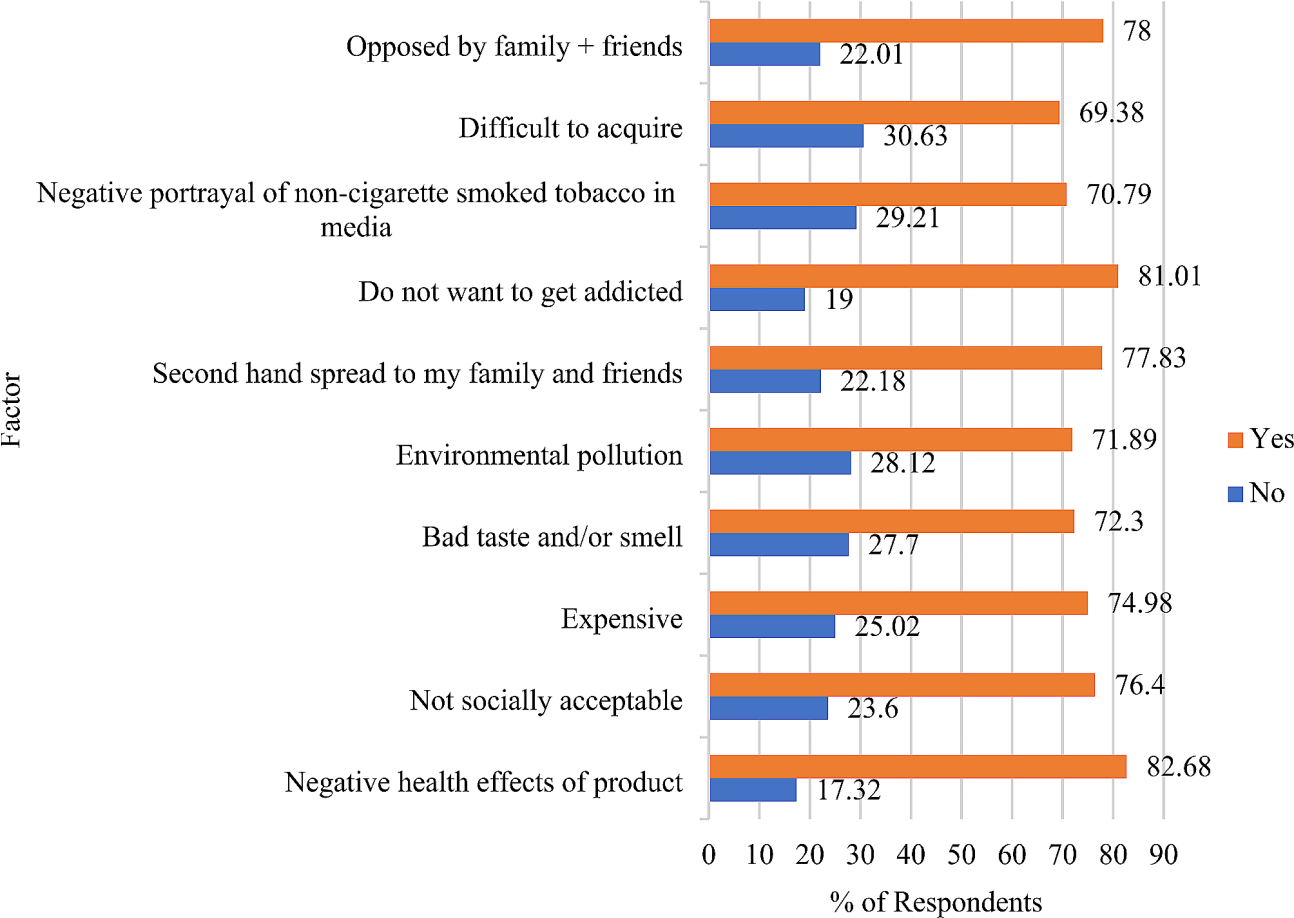
Factors deterring HTP use among respondents

Figure 3 shows the factors deterring respondents from using HTPs. The most selected deterrent were the negative health effects of the product (82.68%) and then the desire to not develop an addiction (81.01%). The least selected deterrents included the bad taste and/or smell (71.89%) and difficulty in acquiring (69.38%).

A total of 16.90% of respondents reported having used HTPs at least once, while the majority (83.10%) had not. Among those who had used HTPs, GLO was the most used product followed by IQOS and the current spending habits varied amongst users, 70 participants, the majority, spent between Rs 4000–6000. In addition, both active users and past users used it mostly on a monthly basis (74.5% and 58.5%, respectively). Regarding future intentions, 52.9% reported that they do not plan to continue using HTPs. Usage patterns of HTPs are depicted in Table 4.

**Table 4:** Usage of HTPs

Variable				*N*(%)
**Have you ever used any of these products, even one time?**				*N* = 1195
Yes				202 (16.90)
No				993 (83.10)
**Among participants who have used at least once (N = 202)**				
**How much do you currently spend on it or have spent on it?**				
Less than Rs 2000				37(18.31)
Rs. 2000–4000				39(19.31)
Rs. 4000–6000				70(34.65)
More than Rs. 6000				56(27.72)
**What type of HTP have you used?**				
GLO				68(33.66)
IQOS				61(30.20)
PLOOM TECH				48(23.76)
Other				25(12.38)
**Do you currently use HTPs?**				
**Yes**				161(79.7)
**No**				41(20.3)
**Currently Active Users (N = 161)**		**Past Users (N = 41)**		
**How often do you use HTPs currently?**		**How often have you used HTPs in the past?**		
Daily	18(11.19)	Daily	6(14.63)	
Weekly	23(14.29)	Weekly	11(26.83)	
Monthly	120(74.53)	Monthly	24(58.54)	
**Do you plan to continue using HTPs? (N = 202)**				
Yes				62(30.69)
No				107(52.97)
Maybe				33(16.34)

## Discussion

Using a web-based survey, we have assessed the knowledge, attitudes, and usage of HTPs among the public in Pakistan. Our respondents revealed that 54% of them had heard of HTPs before this survey while only 17% agreed to using it before. Higher income households and females were found to have higher knowledge scores. Among motivators, subjective enjoyability and alternative to cigarettes were significant factors while the most frequently reported deterring factors were negative impact on health and the potential for addiction.

Our results are in line with previous data which suggests a greater use and thereby knowledge of traditional tobacco products compared to HTPs in Pakistan. A similar study in the UK suggested a comparable figure of 34.8% of its citizens having heard of HTPs [30]. Although limited local data from Pakistan hindered a direct comparison, our findings align with global trends and present a comparable estimate of HTP awareness, hinting at the spread of information through globalization. In contexts similar to Pakistan, a recent study conducted in Saudi Arabia, Egypt, Kuwait, and Yemen reported an awareness rate of 24.2% [31]. Moreover, a study conducted in Poland in 2019, with a participant profile largely consisting of medical students similar to the current study, exhibited a significantly higher awareness rate of 76.5% [32]. This suggests potential variations in awareness levels among different demographic groups. The global trend of increasing knowledge and usage of HTPs [33], particularly among younger adults and men [34], emphasizes the importance of understanding regional disparities and tailoring educational efforts to specific demographics.

When asked about the contents of these products, 50% of the participants believed they contained both nicotine and tobacco, while 27.8% believed they contained only nicotine. A survey among practitioners has revealed similar misconceptions and varied knowledge about nicotine [35]. In addition, a US survey even revealed low awareness regarding the chemicals in tobacco products [36].

Notably, 40.25% thought these products were not prohibited by law and 38.24% were unsure if these products were prohibited by law. Currently, the sale and use of HTPs is not banned in Pakistan but no clear legislation exists regarding HTP use. A few countries however, including India, Panama and Ethiopia have legislated the ban on the sale of HTPs, while Mexico and Turkey have placed a ban on its import [37]. Implementation of tobacco laws is important but awareness of existing legislature guides adherence [38].

Around 50% of respondents exhibited accurate knowledge regarding the ingredients of HTPs, suggesting a moderate level of awareness [39]. This finding could be attributed to a combination of factors, including our cohort comprising of mostly students, in addition to local public health campaigns, media exposure, and educational initiatives at respective institutions aimed at disseminating information about the constituents of tobacco products. However, 28% of respondents did select only nicotine in the product, possibly showing the effectiveness of marketing campaigns for non-cigarette tobacco products which generally rely on an image of reduced health effects compared to traditional cigarettes.

In addition, only 40% correctly identified that HTPs are not prohibited by law in Pakistan, which indicates a substantial gap in regulatory awareness. Despite in recent times, the Ministry of National Health in Pakistan has sought approval for their use [40]. This lack of knowledge might be influenced by limited governmental communication or public discourse on the legal status of HTPs, necessitating improved efforts to convey regulatory information to the public. While a majority (68.4%) recognized the potential serious health risks associated with HTP use, the existence of a greater percentage still unaware of these risks underscores the need for health education campaigns targeting HTP use.

Our study further indicates lesser odds of adequate knowledge regarding HTPs among lower-income households, individuals with fewer years of education and from the province of Balochistan, shedding light on disparities among awareness campaigns, which are likely concentrated in easier to access urban areas.

A substantial proportion of individuals agreed that HTP usage is linked to feelings of guilt and unwellness, while concurrently disagreeing with the notion that it makes one feel happy and young. Emotional associations with HTP usage play a major role in developing public perceptions. These contrasting beliefs may be rooted in evolving societal attitudes toward the consumption of tobacco and alternative products and association of guilt and a sense of unwellness could stem from growing awareness of the potential health risks associated with tobacco use in general [41, 42].

The most reported factor discouraging HTP use among our participants was negative health effects. Negative effects tend to be significant deterrents to use as evidenced in previous studies [43–45]. The Tobacco Products Scientific Advisory Committee of the Food and Drug Administration (FDA) in 2018 voted against endorsing Heated Tobacco Products (HTPs) as carrying reduced harm compared to conventional cigarettes [46]. Similarly, WHO contends that even if exposure to harmful chemicals in HTPs is diminished, it does not confer harmlessness nor does it translate to a reduction in health risks [47]. Although more conclusive evidence is needed on this matter, from a policy viewpoint, a verdict is crucial before allowing its use.

In addition, 75% of participants agreed of HTPs being expensive and this is a predictable result from an LMIC. A study from Korea had similar findings where higher socioeconomic status was associated with HTP usage [48].

Pertinent factors in encouraging the use of HTPs in our respondents included the appeal of various flavors, stress relief properties, and their perceived role as an alternative to traditional cigarettes. A survey conducted in the UK indicated that individuals who engaged in smoking and vaping were more inclined to have experimented with HTPs [30]. This suggests that the desire to find a product aiding in smoking cessation might drive individuals to explore HTPs. The appeal of flavors in HTPs and their stress-relieving properties likely serve as motivators for usage in young populations. Flavors can introduce novelty and variety, addressing taste preferences and potentially masking the harshness associated with traditional tobacco smoke. A prevailing sentiment among participants regarding the necessity for comprehensive research to explore the consequences of using HTPs. The concern stems from the influence of advertisements that often portray HTPs as a safer alternative, potentially shaping the opinions of users.

Despite the multitude of encouraging factors, there exists a low prevalence of current users in our population. A potential barrier could be the cost of the product, with a device and pack of 100 heat sticks selling for approximately PKR 25,000 or more [49]. However, among both past and current users, frequency of use was reported on a weekly to monthly basis, similar to other studies which reported weekly HTP use [8]. This could be due to the less addictive nature of HTPs compared to cigarettes, but further studies are needed to confirm their addictive potential.

## Limitations

Being a web-based survey, responses were limited to the options given, therefore, a follow-up qualitative study is essential to process the subjective perceptions of its use. Participants in survey-based studies may report social desirability bias, besides, because the surveys capture a snapshot at a given time, it does not account for any long-term changes in respondents’ understanding or attitudes. Snowball sampling was also used to collect responses from a diverse population. Moreover, our study was unable to obtain equal representation from all provinces of Pakistan, likely attributable to its web-based nature. Therefore, future studies in Gilgit-Baltistan, Balochistan and Azad Jammu and Kashmir should attempt to conduct face-to face interviews, as it is crucial to obtain a national perspective to influence national level policy.

## Study recommendations

Our study has demonstrated that although there was some awareness with the concept of HTPs in our study population, there was much left to be desired when it came to accurate knowledge regarding these products. Moreover, there was a significant gap in knowledge between certain demographic groupings. Awareness campaigns on the health effects of HTPs are therefore necessary and should be tailored to accommodate those who are more likely to lack knowledge of HTPs. The high percentage of respondents who were unsure of the legal status of HTPs might be a consequence of the absence of any legislation regulating HTP use in Pakistan. As HTP adoption is still in its infancy in the country, legislation targeting HTP use could serve to reduce uptake of these products. With the subjective enjoyment of HTPs being a significant motivator to the pursuit of HTPs in our study population, it is essential to monitor the marketing of HTPs as a lifestyle or leisure product. Instead, legislation should help guide the marketing of these products to be more along the lines of a smoking cessation aid, a proven utility of these products, and another major motivator for HTP use in our study population. Future studies should also attempt to conduct qualitative in-depth interviews for a representative sample across Pakistan, as it is crucial to obtain a national perspective to influence national level policy.

## Conclusion

Our study found that knowledge scores were higher for women and households with greater incomes. The potential for addiction and the negative health impacts were the most often cited deterrents, although subjective enjoyment and a better option than cigarettes were also significant motivators. Our study is the first of its kind to investigate HTPs-related attitudes in the South Asian region. Understanding the public view of HTPs is crucial for future interventions to inform public understanding of tobacco products. Although, HTPs are still a relatively newer product in the Pakistani public’s eyes, pertinent motivators exist for its future use. Our study provides additional key indicators of knowledge, which can be used as a guide to keep the public informed and lists encouraging and deterring factors to gain insight into public opinion. It is recommended that continuous monitoring and public research are crucial to investigate the influence of HTPs as a possible entry to tobacco smoking.

## Data Availability

The study questionnaire has been thoroughly outlined in the methods. Additional datasets and its associated materials are available from the corresponding author upon reasonable request.

## Abbreviations

ERC: Ethical Review Committee
FDA: Food and Drug Administration
HTP: Heated Tobacco Product
